# Protective effects and mechanism of curcumin in animal models of pulmonary fibrosis: a preclinical systematic review and meta-analysis

**DOI:** 10.3389/fphar.2023.1258885

**Published:** 2023-10-13

**Authors:** Fang Hanyu, Hong Zheng, Wang Jiaqi, Dong Tairan, Zhao Yiyuanzi, Yang Qiwen, Liu Ying, Zhang Hongchun, Liu Lu

**Affiliations:** ^1^ Graduate School of Beijing University of Chinese Medicine, Beijing, China; ^2^ Dongzhimen Hospital of Beijing University of Chinese Medicine, Beijing, China; ^3^ The Second Health and Medical Department, China-Japan Friendship Hospital, Beijing, China; ^4^ Department of Traditional Chinese Medicine for Pulmonary Diseases, Center of Respiratory Medicine, China-Japan Friendship Hospital, Beijing, China; ^5^ Department of Traditional Chinese Medicine for Pulmonary Diseases, Jining Hospital of Xiyuan Hospital of China Academy of Chinese Medical Science, Jining, Shandong, China

**Keywords:** pulmonary fibrosis, curcumin, animal models, potential mechanisms, meta-analysis

## Abstract

**Introduction:** At present, there is a lack of effective treatment for pulmonary fibrosis (PF), and a number of studies have confirmed that curcumin (CUR) has a good effect on PF.

**Research Qusetion:** Is CUR effective in preclinical trials for PF and what is its mechanism of action?

**Methods:** Animal reports of PF treated with CUR were searched from Pubmed, Embase, Web of Science and Cochrane Library from 1 January 2000 to 19 April 2023 to compare CUR treatment of PF with a no-intervention model group. A previous registration (nsply registration number: INPLASY202360084) of this review protocol was undertaken.

**Results:** The meta-analysis included 27 publications and 29 studies involving 396 animals. CUR significantly improved the degree of fibrosis, levels of inflammation, and oxidative imbalances in lung tissue in animal models of PF. In terms fibrosis, such as HYP content (SMD = −4.96; 95% CI = −6.05 to −3.87; *p* = 0.000).In terms of inflammatory indicators, such as MPO activity (SMD = −2.12; 95% CI = −4.93 to 0.69; *p* = 0.000). In terms of oxidation index, such as MDA (SMD = −5.63; 95% CI = −9.66 to −1.6; *p* = 0.000).

**Conclusion:** CUR significantly improved the degree of fibrosis, levels of inflammation, and oxidative imbalances in lung tissue in animal models of PF. Due to the quantitative and qualitative limitations of current research, more high-quality studies are needed to verify the above conclusion.

## 1 Introduction

Pulmonary fibrosis (PF), as an end stage pathological manifestation of various lung diseases, is characterized by the damage of lung epithelial cells, the accumulation of fibroblasts and the deposition of extracellular matrix rich in collagen, which is prominent in chronic dry cough and dyspnea caused by exercise (Spagnolo et al., 2015; Hill et al., 2019). Idiopathic pulmonary fibrosis (IPF) is still the most common and serious type of pulmonary fibrosis (Raghu et al., 2019). Its incidence rate, morbidity and mortality are increasing year by year (Dove et al., 2019; Navaratnam and Hubbard, 2019; Wakwaya and Brown, 2019). PF has an extremely poor prognosis, with a median survival period of 3–5 years (Milne et al., 2018; Luppi et al., 2021), and it affects about 3 million people worldwide (M et al., 2017), bringing serious economic burden to patients (Raghu et al., 2004).

In the pathogenesis of PF, existing studies have found that sustained inflammation and oxidative damage are believed to play a major role (Cannito et al., 2010; Kropski et al., 2013), and both can effectively induce epithelial mesenchymal transition (EMT) or abnormally activated fibroblasts. The primary site of lung injury is the interstitium, located between the epithelial and endothelial basement membranes (American Thoracic Society and European Respiratory Society, 2002). Initial injury repairs and rebuilds the tissue under normal conditions, but in IPF impaired healing occurs and abnormal epithelial-mesenchymal interactions are induced (Fernandez and Eickelberg, 2012). It will lead to the secretion of a cascade of pro-inflammatory molecules that activate the proliferation of fibroblasts (King et al., 2011; Fernandez and Eickelberg, 2012). EMT induces fibrotic effects through its upstream signaling pathways, and interfering with or blocking the effector molecules associated with EMT can inhibit the development of pulmonary fibrosis (Hosseinzadeh et al., 2018; Jolly et al., 2018). A large amount of collagen is secreted to repair damaged tissue (You et al., 2015; Gouda and Bhandary, 2018), which leads to excessive deposition of extracellular matrix (ECM) and ultimately leads to the formation of fibrotic lesions (Pardo and Selman, 2002). Pirfenidone and Nintedanib can reduce excessive deposition of matrix collagen and have anti fibrosis effect, so they are approved for first-line treatment (Raghu et al., 2015; Raghu et al., 2022). However, current research shows that these two drugs cannot cure or reverse IPF (Wollin et al., 2015), and have obvious adverse reactions (Galli et al., 2017), as well as the high cost (Teague et al., 2022). But they are still the fundamental treatment options in clinical practice today, so it is crucial to find new treatment methods to prevent and treat PF.

With the development of alternative and complementary medicine, the application of natural herbs has become a new hotspot. Curcumin (1,7-bis - (4-hydroxy-3-methoxyphenyl)—hepta-1,6-diene-3,5-dione) is an acid polyphenol, a diketone compound, which is widely found in the roots of many plants, such as turmeric, zedoary, turmeric, calamus, etc., (Fu et al., 2021; Urošević et al., 2022). Research has shown that CUR can weaken the pathological progression of various fibrosis models, and its anti-fibrosis mechanism may be related to reducing collagen accumulation (Gorabi et al., 2020), such as in studies of oral subcutaneous fibrosis (Ingle, 2020), liver fibrosis (Tawfeek et al., 2023), Renal fibrosis (Wang et al., 2020), etc.

Recent studies have shown that the mechanism of action of CUR in treating PF is mainly through regulating TGF-β signaling pathway, NF- κB signaling pathway, mitogen activated protein kinase (MAPK) signaling pathway, TLRs signaling pathway, and PPARγ/PDGFβ signaling pathway, inhibiting the proliferation and differentiation of fibroblasts, regulating cell apoptosis, inhibiting inflammatory reactions, inhibiting oxidative stress and ECM deposition (Gouda MM. et al., 2020). The TGF-β signaling pathway mediates the regulation of the EMT signaling pathway, and it can be mediated through both smad-dependent and non-smad-dependent pathways (Chitra et al., 2015). Therefore, CUR can target TGF-β to regulate EMT (Tyagi et al., 2019; Shaikh et al., 2021a), and can also block the activation of SMAD and non-SMAD signaling (Shaikh et al., 2020a; Page et al., 2021). However, although the PF animal model is mature and the experimental design is relatively complete, different researchers often focus on different indicators and report differences in the efficacy of the same indicators. Therefore, we have decided to conduct this study.

**FIGURE 1:**
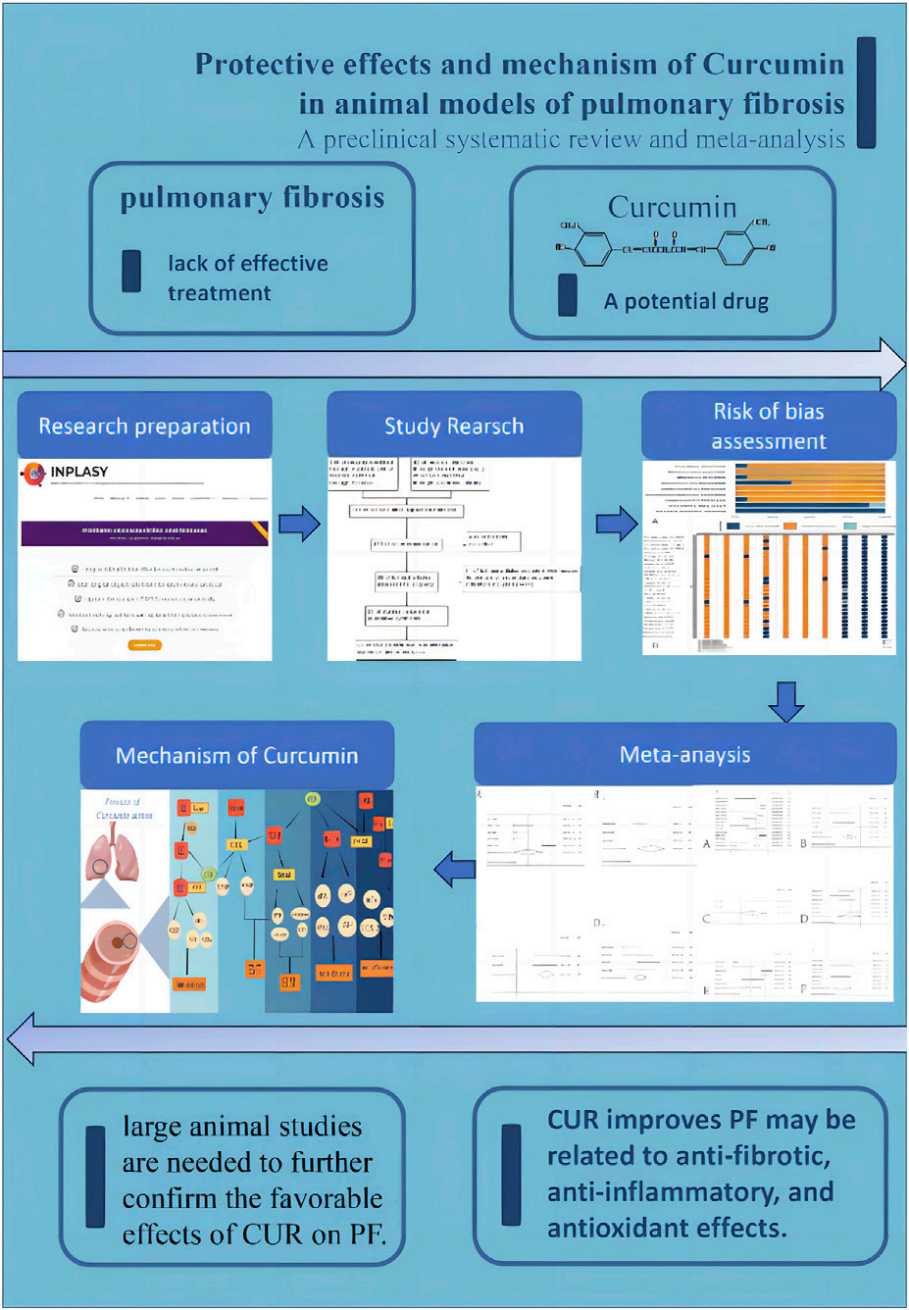
Research roadmap.

We first determine whether CUR can reverse PF, assuming it is positive. We believe that CUR is a potential anti fibrotic drug that needs to be developed for further research and has clinical significance. However, the treatment of PF in animals by CUR remains uncertain and lacks sufficient evaluation, so it is necessary to conduct systematic evidence-based evaluation. Our goal is to systematically evaluate and meta-analyze the effectiveness and safety of CUR in treating PF in animal models, to identify the relevant factors of CUR in the PF process, and to further explore its cellular and molecular mechanisms. A research roadmap is shown in Figure 1.

## 2 Study design and methods

### 2.1 International prospective register of systematic reviews registration

This review is designed and implemented according to the Systematic Review and Meta-Analysis (PRISMA) Preferred reporting project (Chitra et al., 2015), which can be reviewed against the published protocol (INPLASY202360084). (See Annex 1 for search information).

### 2.2 Eligibility criteria

The criteria for inclusion in meta-analyses follow the Participant Type, Intervention, Comparison, Outcome and Type of Study (PICOS) framework. Type of participant (P): Included all pure PF animal models without species, sex, and modeling method restrictions, and excluded non-PF animal models and other disease-related PF animal experiments. Type of intervention (I): Included any type of CUR intervention, regardless of dose, platform of administration, route of administration, time of administration, or duration, and CUR was not the only intervention. While CUR analogues were excluded. Comparison (C): The model group was modeled only and received the same amount of non-functional substances (normal saline or distilled water) or received no intervention. No control group was excluded from the study. Result type (O): No predetermined result indicator was excluded. Type of study (S): All controlled studies of the administration of CUR in PF animals, reviews, conference abstracts, *in vitro*, human, or clinical studies were not considered.

### 2.3 Outcomes

We had the primary outcome: hydroxyproline (HYP) content. The secondary outcomes were TGF-β concentration, myeloperoxidase (MPO) activity, tumor necrosis factor (TNF)-α concentration, malondialdehyde (MDA), NO, GSH.

### 2.4 Information source and search strategy

To identify animal reports on the use of CUR for PF, we searched the following electronic literature databases from 1 January 2000 to 19 April 2023, Pubmed, Embase, Web of Science, and the Cochrane Library. Searches of PubMed database mainly used a combination of MeSH and free text terms to identify diseases, interventional drugs, and animals, and the search algorithm adjusted the search terms according to different database requirements. Specific 4 database search strategies are described in the Supplementary Materials.

### 2.5 Study selection

Two reviewers independently conducted initial screening based on titles and abstracts, then the full text of potentially eligible articles was screened and finalized, with any disagreements resolved through discussion and negotiation, before the other two reviewers scrutinized the data.

### 2.6 Extraction and analysis

Two reviewers independently extracted data manually from the included literature, and the Excel table included the following items: 1) Publication details: first author’s last name and year of publication. 2) Details of the animal (sample size, species, age, sex, weight, and PF animal model). 3) Treatment information: source, concentration, route of administration, delivery system, duration of intervention, and dose of CUR. 4) Observation indicators: All outcome indicators were continuous data, so the mean values and standard deviations of the control group and the intervention group were extracted. If there are several doses or intervention time available in an experiment, the highest dose or the longest intervention duration should be selected for analysis in order to better reflect the characteristics of CUR action. If there are multiple sample sizes for the same outcome indicator, the minimum sample size will be selected for analysis in order to reduce the risk of bias. When the raw data is only available in the graph, the GetData Graph Digitization software (version 2.26) will be used to estimate the data.

### 2.7 Risk-of-bias assessment

Quality assessment was carried out independently by two reviewers and any differences were resolved through discussion, negotiation, and discussion with the third reviewer. The Center for Systematic Review of Laboratory Animal Studies (SYRCLE) Risk of Bias (RoB) Tool, a 10-item scale was used to assess the quality of included animal studies, indicated by “yes”, “No” and “unclear” when the assessment results were “low,” “high” and “unclear.”

### 2.8 Data synthesis and analysis

Statistical analysis data from all included studies were summarized using Revman 5.3 and Stata 16.0. All results were continuous variables, so we used the standard mean difference (SMD) of 95% confidence intervals (CIs) to represent the intervention effect. The heterogeneity between the study and the subgroup was evaluated using I^2^ statistics. When I^2^ ≤ 50%, the heterogeneity of the included study was small, and the fixed-effect model was used for analysis. And when I^2^> 50%, a random effects model was used for meta-analysis, while subgroup analysis and sensitivity analysis were used to investigate sources of heterogeneity. If the results were at least 10 animal trials, publication bias was assessed using funnel plots, Egger tests, and Begg tests.

## 3 Results

### 3.1 Inclusion of study selection

**FIGURE 2:**
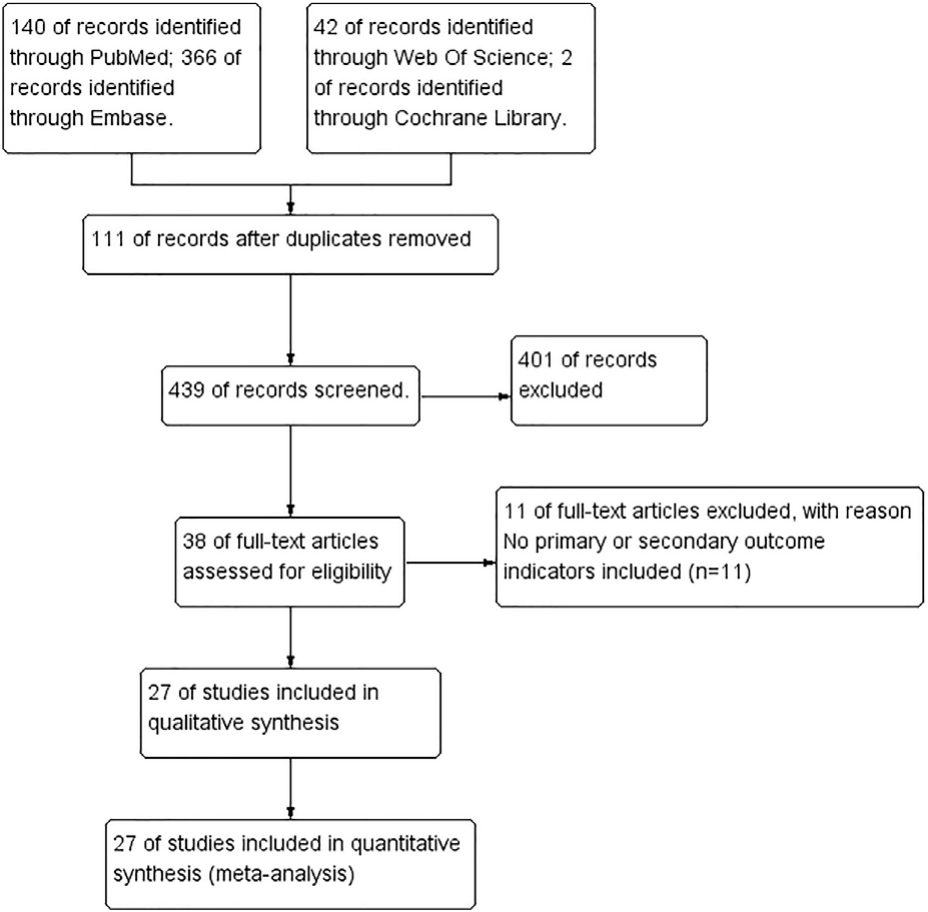
Preferred Reporting Items for Systematic Reviews and Meta-analyses flow chart showing included and excluded trials.

Figure 2 is a flow chart of search, inclusion and exclusion through a preliminary search of the following four electronic databases: PubMed, Web of Science, Embase and Cochrane Library, we found 550 studies and 439 studies remained after removing duplications. After reading the titles, abstracts and full text, 401 studies were excluded, and a total of 38 studies met the inclusion criteria. Eleven studies (Xu et al., 2007; Chen et al., 2008; Zhang et al., 2011; Avasarala et al., 2013; Chen et al., 2018; Gouda and Bhandary, 2018; Shaikh et al., 2020b; Durairaj et al., 2020; Shaikh and Prabhakar Bhandary, 2020; Shaikh et al., 2021b; Fathimath Muneesa et al., 2022) which did not include primary or secondary outcome measures were excluded. In the end, there were 27 studies were included (Punithavathi et al., 2000; Venkatesan, 2000; Punithavathi et al., 2003; Punithavathi et al., 2006; Zhou et al., 2006; Zhang et al., 2007; Zhao et al., 2008; Jiang et al., 2009; Lee et al., 2010; Smith et al., 2010; Hamdy et al., 2012; Cho et al., 2013; Tyagi et al., 2014; Banerjee et al., 2015; Li et al., 2015; Kar et al., 2016; Tyagi et al., 2016; Chen et al., 2017; Hu Y. et al., 2018; Chen et al., 2019; Gouda M. M. et al., 2020; Barsan et al., 2021; Durairaj et al., 2021; Hemmati et al., 2021; Hosseini et al., 2021; Miao et al., 2021; Kumari and Singh, 2022), two of them (Smith et al., 2010; Miao et al., 2021) were included in this meta-analysis, each of which included two independent experiments, so this study included a total of 27 studies and 29 independent experiments.

### 3.2 Trial characteristics

We present the characteristics of the included studies in Annex 1. A total of 396 experimental animals (201 in the intervention group and 195 in the control group) were included in this meta-analysis. All publications were published between 2000 and 2022, and most of the research was conducted in China (*n* = 9) and India (*n* = 10). Sample sizes ranged from 6 to 35. Wistar rats, Sprague—Dawley rats, BALB/c mice and C57BL/6 mice were preferred. Rat weight fluctuated between 160 and 350 g and mice weight fluctuated between 20 and 30 g. Of the studies detailing the sex of the animals, female (*n* = 3), male (*n* = 18), and female half and half (*n* = 1), six did not report sex and only seven reported the age of the animals in detail.

All studies controlled the dose of CUR between 30 and 1000 mg/kg, but the dose varied widely. Researchers preferred gastric tube administration (*n* = 9) and intraperitoneal injection (*n* = 5). CUR carriers or mediators were reported in 13 studies, and the source of CUR was reported in 20 studies, and 12 of them were provided by Sigma-Aldrich, United States. In terms of time point of drug intervention, each independent experiment selected prevention (*n* = 2), treatment (*n* = 19), and combination of prevention and treatment (*n* = 8) for intervention, respectively. In terms of modeling methods, bleomycin (BLM) (*n* = 13), paraquat (PQ) (*n* = 7), and silica (*n* = 4) had the largest selection of independent experiments.

### 3.3 Risk of bias results

**FIGURE 3:**
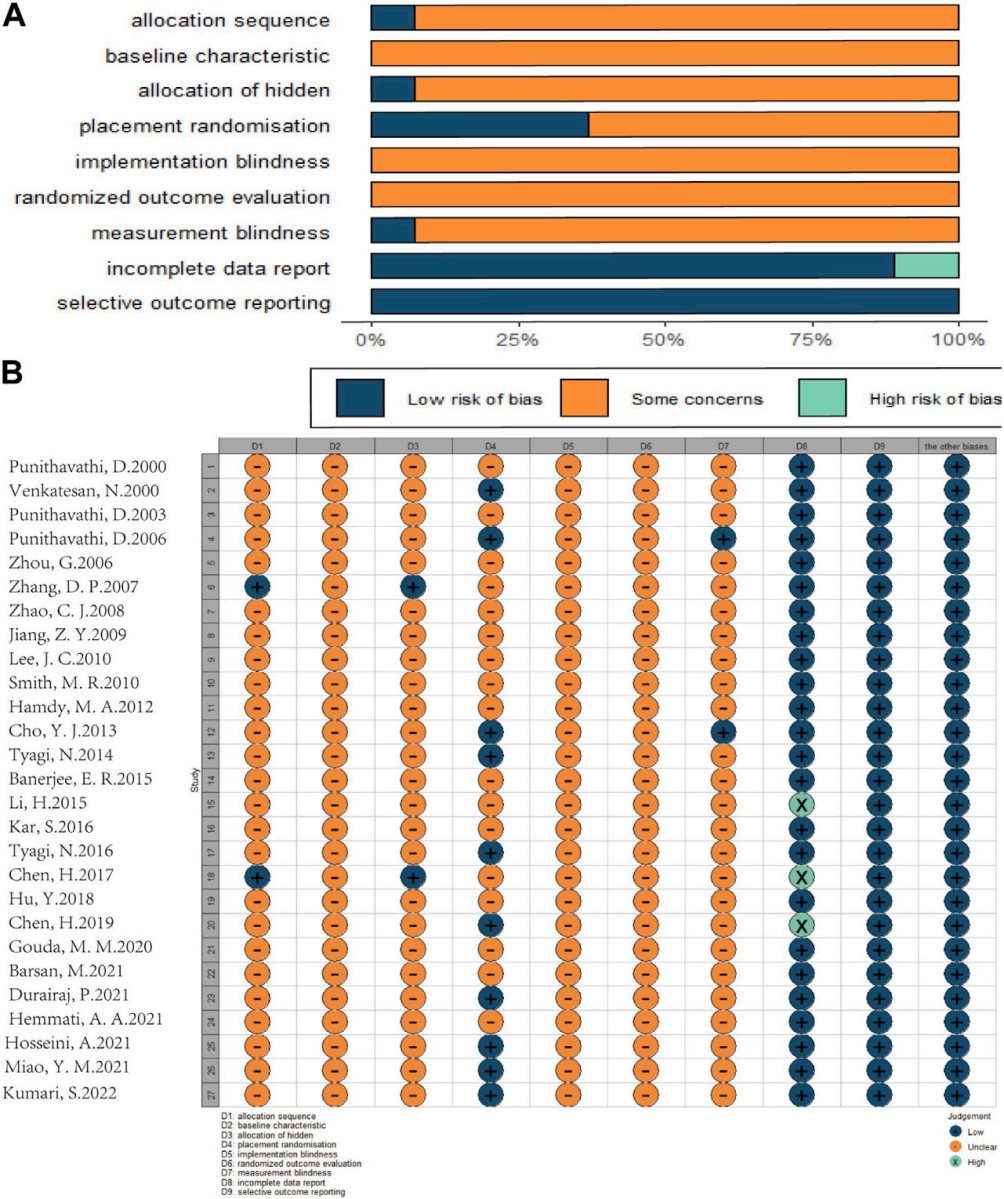
Risk of bias assessment table as following. Assessment of literature quality results obtained through the risk of bias by SYRCLE based on Cochrane tools. **(A)** Risk of bias diagram; overview of authors’ judgments for each risk of bias item, expressed as a percentage of all included studies. **(B)** Risk of bias summary diagram; review of authors’ judgments for each risk of bias item for each included study.

According to SYRCLE’s Risk of Bias tool, the risk of bias and quality of all included studies were assessed. The evaluation results are as follows: 2 studies (Zhang et al., 2007; Chen et al., 2017) were identified as “low risk” because they used a random number table, while the remaining 25 studies were identified as “uncertain” in terms of random sequence generation and allocation of different groups. Among them, 12 studies (Zhou et al., 2006; Zhao et al., 2008; Jiang et al., 2009; Cho et al., 2013; Tyagi et al., 2014; Li et al., 2015; Kar et al., 2016; Tyagi et al., 2016; Chen et al., 2019; Hemmati et al., 2021; Hosseini et al., 2021; Kumari and Singh, 2022) only described “randomization” without explaining the randomization method, and the rest of the studies lacked explanation on this aspect. Not all studies reported whether the distribution of relevant baseline levels was balanced between the experimental and control groups, so the “baseline characteristics” were “uncertain;” 10 studies (Venkatesan, 2000; Punithavathi et al., 2006; Cho et al., 2013; Tyagi et al., 2014; Tyagi et al., 2016; Chen et al., 2019; Durairaj et al., 2021; Hosseini et al., 2021; Miao et al., 2021; Kumari and Singh, 2022) were rated as low risk because they detailed the consistency and randomness of the conditions and environment in which laboratory animals were kept; None of the included studies mentioned blinding animals during the implementation of the intervention; 7 studies (Zhou et al., 2006; Zhao et al., 2008; Smith et al., 2010; Tyagi et al., 2014; Chen et al., 2017; Chen et al., 2019; Kumari and Singh, 2022) only described the “randomized” selection of experimental animals for result evaluation, without explaining the randomization method, and the other studies did not explain this aspect. In the measurement of results, only 2 studies (Punithavathi et al., 2006; Cho et al., 2013) reported the blinding of surveyors and were identified as “low risk,” while the other studies were identified as “uncertain.” 3 studies (Li et al., 2015; Chen et al., 2017; Chen et al., 2019) were rated as high risk for “loss of follow-up bias,” because they had animal death phenomenon but did not explain whether the missing data affected the authenticity of the results, and the other studies were rated as “low risk.” All studies were rated as “low risk” when assessing “selective reporting bias” and other biases (Figure 3).

### 3.4 Primary outcome: hydroxyproline (HYP) content

**FIGURE 4:**
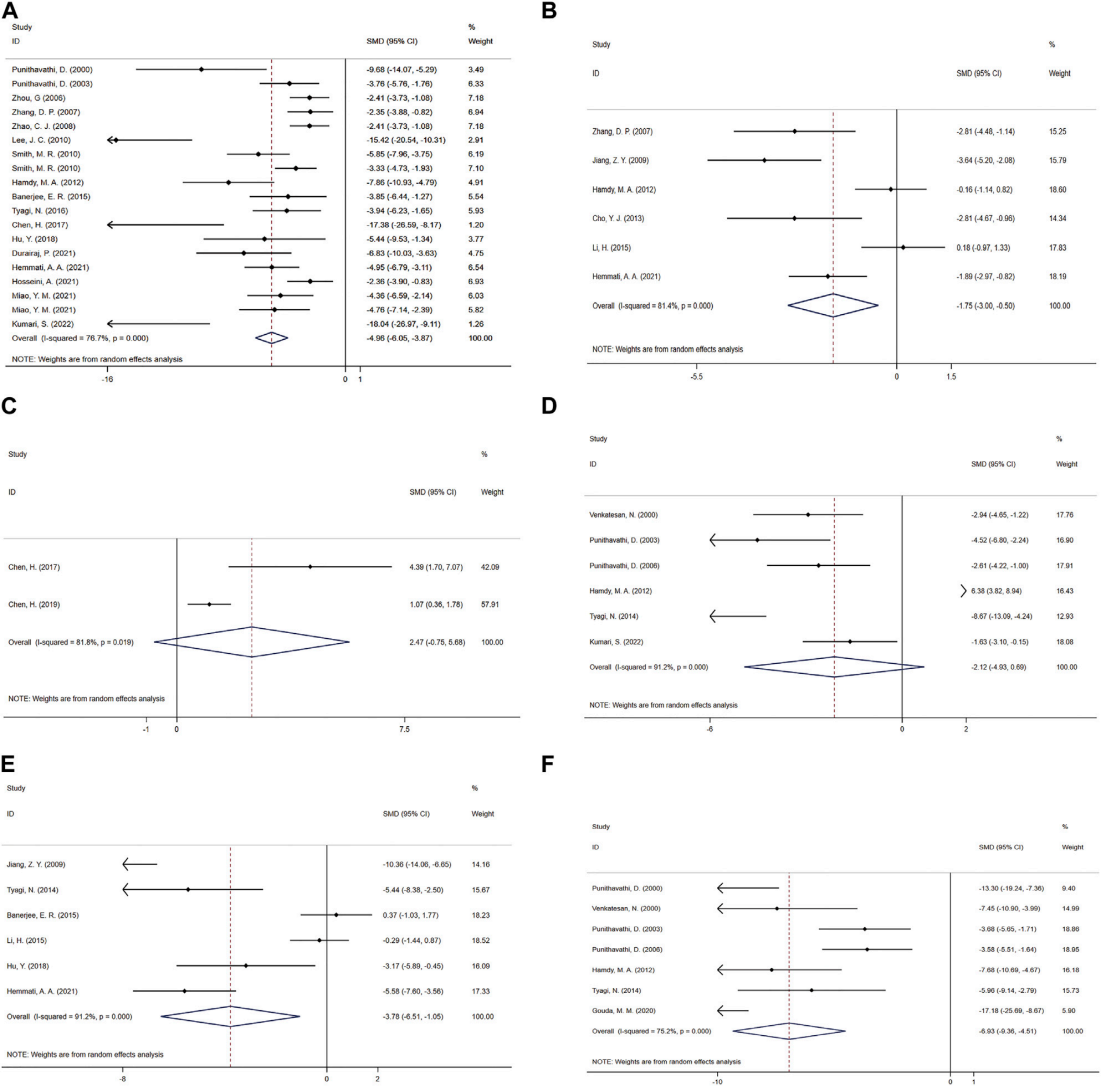
Effect of CUR on Fibrosis and inflammation indicators in PF animals. **(A)** Forest plot of HYP content. **(B)** Forest plot of TGF-β concentration. **(C)** Forest plot of PaO2. **(D)** Forest plot of MPO activity. **(E)** Forest plot of TNF-α concentration. **(F)** Forest plot of BALF protein content.

Forest maps were created based on 19 independent experiments from 17 studies (Punithavathi et al., 2000; Punithavathi et al., 2003; Zhou et al., 2006; Zhang et al., 2007; Zhao et al., 2008; Lee et al., 2010; Smith et al., 2010; Hamdy et al., 2012; Banerjee et al., 2015; Tyagi et al., 2016; Chen et al., 2017; Hu Y. et al., 2018; Durairaj et al., 2021; Hemmati et al., 2021; Hosseini et al., 2021; Miao et al., 2021; Kumari and Singh, 2022). Only one of them originated from bronchoalveolar lavage fluid (BALF) (Durairaj et al., 2021) and all others were from lung tissue showed that: In comparison with the control group, CUR significantly reduced HYP content (SMD = −4.96; 95% CI = −6.05 to −3.87; heterogeneity χ2 = 77.37, df = 18, *p* = 0.000, I^2^ = 76.7%, Figure 4A). To explore the heterogeneity among the studies, the results of meta regression showed: The effect size of HYP content was correlated with the choice of intervention time (t = −3.52, *p* = 0.004). Animal model of type (t = −0.32, *p* = 0.751), animal sex (t = −0.04, *p* = 0.966), dosing mode (t = −1.36, *p* = 0.197), treatment time (t = 2.13, *p* = 0.055), and animal models in model selection (t = −1.2, *p* = 0.253) were irrelevant. According to the pre-determined subgroup analysis: In animal experiments, Wistar rats (*p* = 0.000), female (*p* = 0.000), gastric intubation (*p* = 0.000), Pre-treatment combined treatment (*p* = 0.003), and more beneficial effects were observed in less than 7 days of treatment (*p* = 0.020) and treatment of SiO2 models (*p* = 0.004).

### 3.5 Secondary outcome

#### 3.5.1 PF related indicators

##### 3.5.1.1 TGF-β concentration

A meta-analysis was conducted for the effect sizes of TGF-β concentrations from six studies (Zhang et al., 2007; Jiang et al., 2009; Hamdy et al., 2012; Cho et al., 2013; Li et al., 2015; Hemmati et al., 2021) (Figure 4B). One was from BALF (Zhang et al., 2007), two from serum (Cho et al., 2013; Li et al., 2015) and the rest were from lung tissue. In view of the significant heterogeneity among the included studies (heterogeneity χ2 = 26.93, df = 5, *p* = 0.000, I^2^ = 81.4%), a random-effects model was selected, and CUR treatment had a significant effect on TGF-β concentration (SMD = −1.75; 95% CI = −3 to −0.5).

##### 3.5.1.2 PaO2

The PaO2 study included a total of two studies (Chen et al., 2017; Chen et al., 2019). Given the significant heterogeneity among the included studies, the efficacy of CUR was significant using random-effects analysis combined with effect size (SMD = 2.47; 95% CI = −0.75 to 5.68; heterogeneity χ2 = 5.49, df = 1, *p* = 0.019, I^2^ = 81.8%, Figure 4C).

#### 3.5.2 Inflammatory index

##### 3.5.2.1 Lung MPO activity

Six studies (Venkatesan, 2000; Punithavathi et al., 2003; Punithavathi et al., 2006; Hamdy et al., 2012; Tyagi et al., 2014; Kumari and Singh, 2022) combined effect sizes of MPO activity, using a fixed-effect model compared to the control group, and CUR improved MPO activity (SMD = −2.12; 95% CI = −4.93 to 0.69; heterogeneity χ2 = 56.56, df = 5, *p* = 0.000, I^2^ = 91.2%, Figure 4D).

##### 3.5.2.2 TNF-α concentration

As for the effect on TNF-α concentration, a paired comparison of six studies (Jiang et al., 2009; Tyagi et al., 2014; Banerjee et al., 2015; Li et al., 2015; Hu Y. et al., 2018; Hemmati et al., 2021) mentioned the effect of CUR on this result. One was from BALF (Hu Y. et al., 2018), two from lung tissue (Jiang et al., 2009; Hemmati et al., 2021) and the rest were from serum. Pooled effect sizes showed that CUR significantly reduced TNF-α concentrations (SMD = −3.78; 95% CI = −6.51 to −1.05; heterogeneity χ2 = 56.56, df = 5, *p* = 0.000, I^2^ = 91.2%, as shown in Figure 4E).

##### 3.5.2.3 BALF protein content

The effect size of BALF protein content in 7 studies (Punithavathi et al., 2000; Venkatesan, 2000; Punithavathi et al., 2003; Punithavathi et al., 2006; Hamdy et al., 2012; Tyagi et al., 2014; Gouda M. M. et al., 2020) was summarized and the forest Figure (Figure 4F) showed that CUR had a significant effect on NO (SMD = −6.93; 95% CI = −9.36 to −4.51; heterogeneity χ2 = 24.17, df = 6, *p* = 0.000, I^2^ = 75.2%).

#### 3.5.3 Oxidation index

##### 3.5.3.1 Lung MDA

**FIGURE 5:**
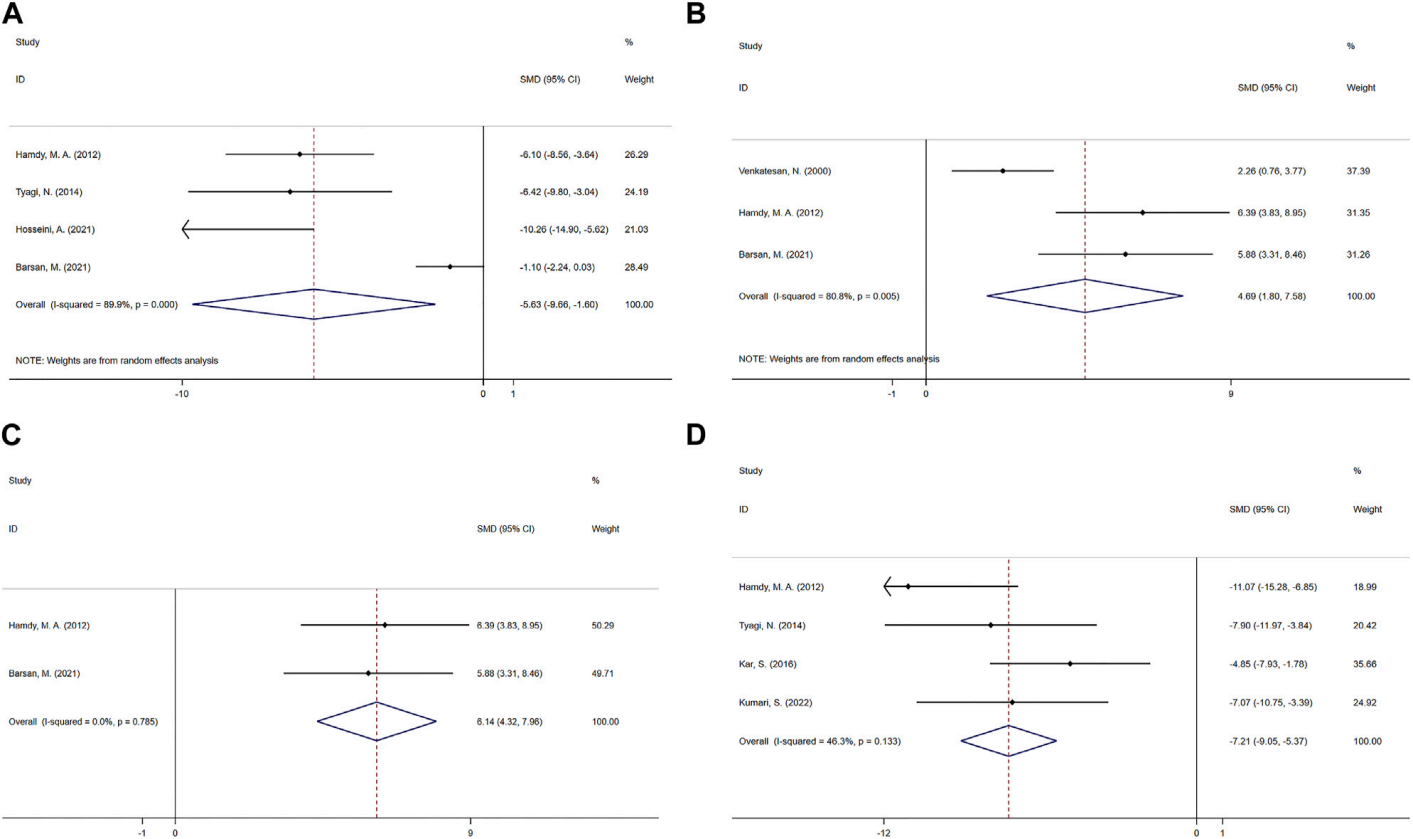
Effect of CUR on oxidation indicators in PF animals. **(A)** Forest plot of MDA. **(B–C)** Forest plot of GSH. **(D)** Forest plot of NO.

The MDA effect size was pooled from a paired comparison of five studies (Zhou et al., 2006; Hamdy et al., 2012; Tyagi et al., 2014; Barsan et al., 2021; Hosseini et al., 2021), and the CUR effect was significant in the random-effects model analysis (SMD = −5.63; 95% CI = −9.66 to −1.60; heterogeneity χ2 = 30.05, df = 3, *p* = 0.000, I^2^ = 89.9%, Figure 5A).

##### 3.5.3.2 GSH

The effect size of GSH was combined from three studies (Venkatesan, 2000; Hamdy et al., 2012; Barsan et al., 2021), and the results were shown in Figure 5B. One was from BALF (Hamdy et al., 2012), one from lung tissue (Hamdy et al., 2012), one from serum (Barsan et al., 2021). Compared with the control group, CUR had a significant improvement effect on GSH (SMD = 4.69; 95% CI = 1.80 to 7.58; heterogeneity χ2 = 10.44, df = 2, *p* = 0.005, I^2^ = 80.8%). Sensitivity analysis showed that excluding Venkatesan, N.2000, the heterogeneity decreased from 80.8% to 0%, and the fixed-effect model shows SMD = 6.14; 95% CI = 4.32 to 7.96; heterogeneity χ2 = 0.07, df = 1, *p* = 0.785, I^2^ = 0%, Figure 5C.

##### 3.5.3.3 NO

Four paired comparisons (Hamdy et al., 2012; Tyagi et al., 2014; Kar et al., 2016; Kumari and Singh, 2022) reported the effect of CUR on NO, one was from BALF (Kumari and Singh, 2022), two from serum (Tyagi et al., 2014; Kar et al., 2016) and the rest were from lung tissue. And the fixed-effect model showed a significant reduction in CUR compared to the control group (SMD = −7.21; 95% CI = −9.05 to −5.37; heterogeneity χ2 = 5.59, df = 3, *p* = 0.133, I^2^ = 46.3%, Figure 5D).

### 3.6 Sensitivity analyses

For outcome measures other than GSH, we performed sensitivity analysis by omitting data from one independent experiment at a time and calculated aggregate data from the remaining studies to demonstrate that the results of all the remaining outcome measures were robust. (See Annex part 2).

### 3.7 Subgroup analysis

Due to the high heterogeneity among studies, we evaluated relevant indicators in terms of animal species, animal sex, dosing mode, intervention time selection, treatment time, and PF model selection. The results showed that animal species and route of administration may be the heterogeneous sources of HYP content. (See Annex part3).

### 3.8 Publication bias

In order to further explore publication bias and heterogeneity, egger and begg tests were carried out for HYP content. Egger: *p* = 0.000, t = −8.54; begg: *p* = 0.001, z = 4.16, the results showed that there was publication bias in HYP content. (See Annex part 4).

## 4 Discussion

PF is characterized by diffuse inflammatory injury and destruction of tissue structure and is an irreversible end-stage disease with most pulmonary interstitial changes. Commonly used clinical drugs are pirfenidone and Nidanib, which can alleviate the progression of PF to a certain extent, but its long-term positive effects have not been confirmed. The pathogenesis of PF remains to be further clarified (Wilson and Wynn, 2009; Spagnolo et al., 2021), and no animal model can fully reproduce the pathological conditions of humans. How to translate the obtained results into further clinical studies is an ongoing challenge. Systematic reviews and meta-analyses of animal studies are considered a valuable tool that can provide important insights into the validity of animal studies, and to improve the precision of estimated effects, which ultimately supports further generalization to human clinical trials.

### 4.1 Summary of research evidence

To our knowledge, no previous meta-analysis has quantitatively assessed the efficacy of CUR in the treatment of PF. In the meta-analysis, which included 27 publications and 29 studies involving 396 animals and was of low methodological quality overall, CUR significantly improved the degree of fibrosis, levels of inflammation, and oxidative imbalances in lung tissue in animal models of PF. This evidence suggests that CUR acts as an antifibrotic agent in animal models of PF through several mechanisms. These findings can provide scientific basis for clinical trials of CUR in the treatment of PF.

### 4.2 Heterogeneity

We further performed subgroup analysis and meta regression for the main outcome HYP content. The results of subgroup analysis and meta-regression indicated that animal species and route of administration may be the source of heterogeneity of HYP content. However, due to the small sample size, these results should be interpreted with caution and more studies are needed to provide precise evidence. Animal sex, intervention time selection, course of treatment, and PF model selection may not be sources of inter-study heterogeneity. Therefore, we speculate that heterogeneity may arise from other differences in the studies, including the dose of CUR, the source of the sample, the modeling method, etc. In addition, we conducted sensitivity analysis on all the results, and the results did not change significantly, indicating that the results were stable.

### 4.3 Interpretation

This meta-analysis did not limit the type and sex of experimental animals. The included animals were mice and rats. Through subgroup analysis, we found that animal species was one of the sources of heterogeneity, and the effect on HYP content was the greatest in Wistar rats. Considering that different models have different mechanisms and sensitivities to PF, further research is needed.

This meta-analysis did not limit the types of modeling. BLM was found in clinical application to induce PF in patients, but this model did not replicate the two characteristics of slow and irreversible progression of human IPF (Li et al., 2022). The advantage of silica model is that silica particles in the lungs are difficult to remove, which can form a persistent stimulus (Degryse and Lawson, 2011). Through subgroup analysis, we found that the CUR model has the greatest effect on HYP content. The inclusion of animal models including radiation, amiodarone, cyclophosphamide, etc., can compensate for the limitations of the PF model to a certain extent, and make a more comprehensive response to the therapeutic effect of CUR in treating PF.

This meta-analysis did not restrict the route of administration for CUR, and meta-regression and subgroup analysis indicated that the route of administration might be the source of heterogeneity in HYP content, and CUR achieved better effect size in HYP through gastric intubation intervention. In view of the considerable differences in the absorption rate and bioavailability of CUR by different routes of administration, as well as the fact that we do not know the conversion relationship between different routes of administration, dose-related subgroup analysis and meta regression were not performed in this meta-analysis. We carefully read all the included studies to clarify the effect of dose versus effect size.

In the PF model, the first 7 days were dominated by alveolitis, and the inflammation decreased and gradually produced extensive fibrosis in the later stage. Subgroup analysis showed that treatment of less than 7 days had a better effect on HYP content, and the effect in this case may reflect more anti-inflammatory effects by blocking the early response. For the choice of intervention time, pretreatment combined therapy achieved better efficacy in HYP content.

Based on the above findings, we suggest that in the treatment of PF, more attention should be paid to the choice of administration mode, dose, administration time and intervention time of CUR to determine the best intervention mode and course of treatment.

### 4.4 Influence and mechanism of CUR on PF animal model

#### 4.4.1 Antifibrotic effect

PF is accompanied by abnormal protein decomposition and proliferation activities in the lungs. In fibrotic pulmonary diseases, the composition and deposition of extracellular matrix (ECM) proteins, including collagen and glycoprotein, are changed (Mei et al., 2021). With CUR’s intervention, lung collagen synthesis and deposition, collagenase and collagenolytic enzyme levels were all reduced, and fibrotic deposition was prevented by regulating collagen turnover, assembly, and deposition in rat lungs (Durairaj et al., 2021). HYP is the main component of collagen and can indirectly reflect the overall collagen deposition (Chang et al., 2017). The balance between Matrix metalloproteinase (MMP)-9 and MMP-2/tissue inhibitor of metalloproteinase (TIMP)-1 is closely related to ECM (Cui et al., 2017; Cabral-Pacheco et al., 2020). CUR can play an anti-fiber role by regulating the expression of MMP and TIMP-related proteins (Tyagi et al., 2016; Hu Y. et al., 2018; Shaikh et al., 2021b; Kumari and Singh, 2022). Since glycoproteins maintain the structural stability of collagen fibers and are resistant to proteolytic enzymes, CUR has the ability to inhibit enhanced deposition of glycoproteins in fibrotic lungs (Zhou et al., 2006). CUR not only inhibits the expression of alpha-smooth muscle actin, but also blocks proliferation in human lung fibroblast cell lines, with its anti-proliferative effects reflecting the blocking of cell cycle progression (Smith et al., 2010). The antifibrotic effect of CUR is associated with inhibition of overexpression of cathepsins K and L (Punithavathi et al., 2003) and mediation of the p53-fibrinolytic system (Teague et al., 2022).

The mediators of fibrotic process include fibrotic cytokines and TGF-β/small mothers against decapentaplegic (Smad) signaling pathway, which can induce cell differentiation, migration, invasion, or proliferative changes and promote ECM deposition. It plays an important role in the process of PF (Hu H. H. et al., 2018; Inui et al., 2021), and CUR therapy protects BLM activation on macrophages by inhibiting the release of TGF-β1 (Durairaj et al., 2021). With the intervention of CUR, the expression of Epithelial Growth Factor Receptor (EGFR) and Proliferative Protein (Ki 67) can be adjusted (Zhao et al., 2008). Mitogen activated protein kinases (MAPK)/extracellular signal regulated kinase (ERK) is an effective target for anti-fibrosis therapy (Chang et al., 2022). The CUR can regulate PF progression through this signaling pathway (Zhang et al., 2007). The effect of CUR also involves the intestinal pathway, which significantly increases the expression of enteric-derived hepatocyte growth factor (HGF) in colon tissue and lung. CUR promotes the expression of HGF in colon fibroblasts and macrophages by inducing 15d-PGJ2 to activate Peroxidase proliferator activated receptor γ(PPARγ) and cAMP response element (CREB), and HGF enters the lung to produce anti-PF effect (Miao et al., 2021).

This meta-analysis confirmed that CUR significantly improved PF levels, including decreased HYP content and TGF-β concentration, and improved PaO2.

As alveolar fibers are heavily replaced in patients with PF, resulting in no gas exchange function, degradation of normal lung tissue structure, and partial loss of lung function, PaO2 can be used to evaluate the presence of symptoms such as hypoxia (Hoiland et al., 2016).

#### 4.4.2 Anti-inflammatory effect

Inflammation is considered to be a pathological factor in the early pathogenesis of PF, and the CUR-mediated anti-inflammatory mechanism in animal models of PF is manifested in the reduction of inflammatory mediators. Increased MPO activity (a marker of neutrophil activation) has been shown to promote fibrosis and advance the progression of end-stage lung disease (Sebastiani et al., 2020; Kishaba, 2022). TNF-α is an inflammatory factor, which can induce the secretion of factors including TGF-β (Sullivan et al., 2009) and MMP (Burrage et al., 2006) and regulate the proliferation of lung fibroblasts (Doherty et al., 2023). BALF protein content, as one of the indicators of lung microenvironment, indirectly indicates the level of lung inflammation and oxidative stress (Wu et al., 2017; Akhter et al., 2022). This meta-analysis confirmed significant improvements in inflammation levels in PF animal models with CUR’s intervention, including reductions in MPO activity, TNF-α concentration, and BALF protein content. Interleukin (IL)-4 and interferon (IFN)-γ are characteristic cytokines of immune Th1 cells and proinflammatory Th2 cells with multiple biological effects, respectively, and play a regulatory role in the proliferation and deposition of fibroblasts and collagen fibers (Ando et al., 1999; Gharaee-Kermani et al., 2001; Yamaji-Kegan et al., 2010; Chang et al., 2021; Rao et al., 2021). In animal models of PF, CUR can inhibit fibrosis by modulating IL-4 (61; 67) and IFN-γ (Zhang et al., 2007; Tyagi et al., 2014; Banerjee et al., 2015; Chen et al., 2019) concentrations.

#### 4.4.3 Antioxidation

**FIGURE 6:**
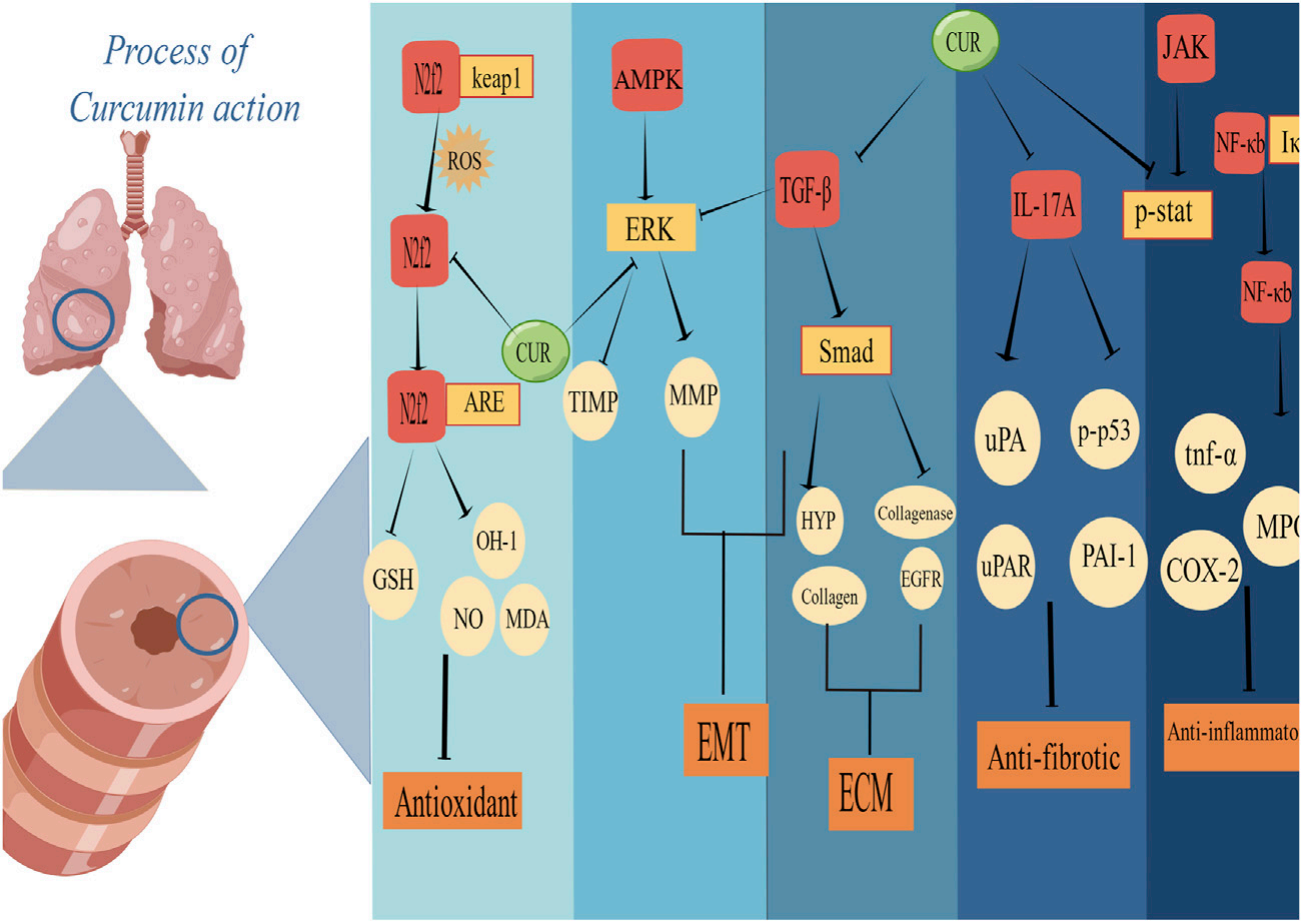
Schematic representation of the possible molecular mechanism of CUR inhibition of PF. (Created using Figdraw.com).

Oxidative stress plays a key role in driving PF. This meta-analysis confirmed that the CUR intervention significantly improved the oxidative imbalance in PF animal models, including decreasing MDA and NO and increasing GSH. Nuclear factor E2 related factor 2(Nrf2) is a key transcription factor that mediates the activation process of heme oxygenase-1(HO-1) to protect cells from oxidative damage, and CUR improves oxidative imbalance by regulating Nrf2/HO-1 gene expression (Lee et al., 2010; Hosseini et al., 2021). CUR ameliorates oxidative imbalances by mediating thiouracil interacting proteins (Ren et al., 2019). MDA is the final product of lipid peroxidation, and its concentration is proportional to the cell damage caused by reactive oxygen species (ROS) (Peng et al., 2022). GSH is essential to protect mercaptan and other nucleophilic groups in proteins from toxic oxygen free radicals (Lu, 2009). Excess NO may play a harmful role by directly inducing tissue damage and hydrogen peroxide formation (Chen et al., 2021). (Figure 6)

### 4.5 Genetic factor

In our study, we found that more attention was paid to the diagnostic significance and mechanism of genetic genes for PF in modern times. Studies have shown that the prevalence of interstitial abnormalities (ILA) in those related to patients with IPF was 15%. At the same time, a perspective from the PF foundation genetic testing work group indicates that attention should be paid to the clinical significance of genetic diagnosis of PF and proposes that gene sequencing and TL measurement for PF patients are meaningful. This seems to be one of the explanations for heterogeneity, but there is still a lack of relevant scientific research, which also provides ideas and directions for further research.

### 4.6 Security

No side effects were observed in any of the included studies, and further study of the safety of CUR in the treatment of PF is the focus and direction, which will also help to observe and understand the limitations of CUR therapy. Based on included studies, we endeavored to find studies on curcumin in the hope of finding reports of related toxicity or adverse reaction studies. We would like to refine and add to the reports in this area, but there is a lack of various pharmacologic studies suggesting that it is safe to use, and no reports of toxicity/adverse reactions yet. In various animal models and clinical studies, CUR has been shown to be very safe, even at doses as high as 8 or 12 g/day. Based on the above findings, the following two points need to be noted in future studies: First, the association between the dose and intervention time of CUR and adverse reactions needs to be further studied. Second, some researchers do not report adverse effects, which can mislead people to believe that there are no adverse effects. We call on researchers to standardize the reporting of animal experiments, including adverse events.

### 4.7 Limitation

First, the measurement methods used in different laboratories were different, and the measurement units were different. Although we used standardized mean differences to reduce the statistical effect size, we still did not completely eliminate bias, which was an important factor in study quality. Second, although we used meta-regression and pre-specified subgroup analyses, due to the limited sample size and insufficient statistical power of each subgroup, no source of significant heterogeneity could be found, which could result from different animal ages, different routes of administration, and different treatment initiation times. In addition, methodological defects might be the key factor leading to the high heterogeneity among studies. The evaluation data were not original data, but from electronic data calipers, which might cause the error of the results to some extent. Third, none of the studies reported the calculation of sample size, and the number of included studies was relatively small, which might have a large positive bias effect, and the existence of publication bias confirmed that the real effect of CUR could be overestimated. Finally, the lack of data from large animals in our statistical analysis, as they shared more pathophysiological features with humans, might also limit the interpretation and extension of our results. So large animal studies are needed to further confirm the favorable effects of CUR on PF.

## 5 Conclusion

Our study has demonstrated that CUR can partially exert anti-PF effects in animal models, and the mechanism by which CUR improves PF may be related to anti-fibrotic, anti-inflammatory, and antioxidant effects. The results of this meta-analysis must be interpreted and applied with an appropriate degree of caution due to factors such as poor methodological quality of the studies and possible publication bias, which could undermine the validity of a positive outcome. Even so, CUR may be a promising drug to treat PF.

## Data Availability

The original contributions presented in the study are included in the article/Supplementary Material, further inquiries can be directed to the corresponding authors.
