# Supplementary material for: Protective effects and mechanism of curcumin in animal models of pulmonary fibrosis: a preclinical systematic review and meta-analysis
Source: Front Pharmacol. 2023 Oct 13;14:1258885. doi: 10.3389/fphar.2023.1258885 (PMC10613035; doi:10.3389/fphar.2023.1258885)
Supplement: Supplementary file 1 [file DataSheet2.pdf]

Author	Year	Country	Type of animal	Weight	age	Gender	sample size (I/C)		Best intervention options	CUR administration method	CUR vehicles or delivery platforms	CUR sources	Purity Of CUR	Pre-processing(I/C)		Pre-processing time	Moulding methods	Treatment interval	Treatment (I/C)		days of treatment	Pick-up time (from endays of mouldaysing)	outcome
Punithavathi, D.	2000	India	Wistar rats	325-350g	*	*	6	6	CUR 300mg/kg	Gastric intubation	gum acacia	*	*	CUR QD	*	10days	Received one intratracheal instillation of BLM (7.5 U/kg)	*	CUR QD	*	7days	7days or 14days or 28days	BALF protein content(7thD),HYP content(14thD or 28thD)
Venkatesan, N.	2000	India	Wistar rats	250±10g	*	Male	6	6	CUR 300mg/kg	Oral	gum acacia	Sigma-Aldrich,USA	*	CUR QD	*	10days	Received one intraperitoneal injection of PQ (50mg/kg)	*	CUR QID	*	24hours	24hours	MPO activity,BALF protein content,(BALF)GSH
Punithavathi, D.	2003	India	Fischer 344 rats	200-225g	*	Male	6	6	CUR 200mg/kg	Gastric intubation	corn oil	Sigma-Aldrich,USA	*	*	*	*	Received two intratracheal instillation of Amiodarone (6.25mg/kg)	*	CUR QD	*	3days or 35days	3days or 35days	BALF protein content(3rdD),MPO activity(3rdD),HYP content(35thD)
Punithavathi, D.	2006	France	Wistar rats	225-250g	*	Male	6	6	CUR 300mg/kg	*	*	*	*	CUR QD	*	7days	Received one intratracheal instillation of BLM (5mg/kg)	*	CUR QD	*	*	7days	BALF protein content,MPO activity,SOD
Zhou, G.	2006	China	Sprague–Dawley rats	198±7g	*	Male	8	8	CUR 200mg/kg	Gastric intubation	Carboxymethylcellulose	Sichuan Jinyujin Technology Co., Ltd	95.60%	*	*	*	Received one intratracheal instillation of BLM(5mg/kg)	1day	CUR QD	Saline	27days	28days	HYP content
Zhang, D. P.	2007	China	Sprague–Dawley rats	180±20g	*	Male	6	6	CUR 50mg/kg	Intraperitoneal administration	Ethanol+polyethylene glycol	Sigma-Aldrich,USA	*	*	*	*	Received one intratracheal instillation of BLM (5mg/kg)	14days	CUR QD	Ethanol+polyethylene	14days	28days	HYP content,(BALF)TGF-β concentration
Zhao, C. J.	2008	China	Sprague–Dawley rats	198±7g	*	Male	8	8	CUR 200mg/kg	Gastric intubation	Carboxymethylcellulose	Sichuan Jinyujin Technology Co., Ltd	95.60%	*	*	*	Received one intratracheal instillation of BLM (5mg/kg)	1day	CUR QD	Saline	26days	28days	HYP content
Jiang, Z. Y.	2009	China	NIH mice	about 20g	adult	Male	9	9	CUR 200mg/kg	Gastric intubation	Carboxymethylcellulose	Sigma-Aldrich,USA	*	*	*	*	Received one intratracheal instillation of silica (5g/L)	14days	CUR BID	distilled water	28days	42days	(Lung tissue)TNF-α,TGF-β concentration
Lee, J. C.	2010	USA	C57BL/6 mice	*	6-10 weeks old	Female	10	10	Diet including 5% CUR	Oral	*	Sigma-Aldrich,USA	*	Diet including 5% CUR	Normal diet	14days	Received 4 months of radiation	*	Diet including 5% CUR	Normal diet	4monthouss	4monthouss	HYP content
Smith, M. R.	2010	USA	C57BL/6 mice	*	*	*	10	10	CUR 300mg/kg	Intraperitoneal administration	Carboxymethylcellulose	Sigma-Aldrich,USA	*	CUR QD	*	3days	Received one intratracheal instillation of BLM(0.075units)	*	CUR QD	*	21days	21days	HYP content
							10	10						*	*	*		10days	CUR QD	*	11days		
Hamdy, M. A.	2012	Egypt	Albino Wistar rats	180-220g	*	Male	8	8	CUR 200mg/kg	Oral	Carboxymethylcellulose	Sigma-Aldrich,USA	*	CUR QD	*	6days	Received intraperitoneal Administer of CP (150 mg/kg) for 2 consecutive days	0	CUR QD	*	8days	14days	HYP content,TGF-β concentration,BALF protein content,GSH,MDA,NO,MPO activity
Cho, Y. J.	2013	Korea	Sprague–Dawley rats	250-270g	*	Male	5	5	CUR 200mg/kg	Gastric intubation	*	Sigma-Aldrich,USA	*	CUR five times per week	*	7days	Received one single 18-Gy dose of thoracic irradiation for 8 weeks	*	CUR five times per week	*	8weeks	8weeks	(Serum)TGF-β concentration
Tyagi, N.	2014	India	parke's mice	25-27g	8 weeks old	Male	5	5	CUR 5mg/kg	Intraasal administration	Dimethyl Sulphoxide	*	*	CUR ST	DMSO	1hours	Received one intraperitoneal injection of PQ (50mg/kg)	*	*	*	*	48hours	BALF protein content,MPO activity, MDA,SOD,NO,(Serum)TNF-α concentration
Banerjee, E. R.	2015	India	BALB/c mice	*	*	*	4	4	CUR 40μl	Intratracheal injection	*	*	*	*	*	*	Received two BLM at day 0.20μl was administered intranasally and 40μl from intratracheally	7days	CUR QW	*	28days	28days	HYP content,(Serum)TNF-α concentration
Li, H.	2015	China	Wistar rats	265±23g	8 weeks old	Male	7	5	CUR 1000mg/kg	Gastric intubation	*	Sigma-Aldrich,USA	*	*	*	*	Received one gastric intubation of PQ(5mg/kg)	30min	CUR QD	distilled water	21days	21days	(Serum)TNF-α,TGF-β concentration
Kar, S.	2016	India	BALB/c mice	*	*	*	4	4	CUR 2μM/kg	Intratracheal injection	*	*	*	*	*	*	Received two BLM (7.5 U/kg) by administered intranasally and intratracheally	7days	CUR QW	*	21days	28days	(Serum)NO
Tyagi, N.	2016	India	Parke's mice	20-27g	8 weeks old	Male	5	5	CUR 5mg/kg	Intraasal administration	Dimethyl Sulphoxide	*	*	CUR ST	*	1hours	Received one intravenous injection of PQ(50mg/kg)	*	*	*	*	48hours	HYP content
Chen, H.	2017	China	Wistar rats	200±20g	*	Half male and half female	6	3	CUR 200mg/kg	Intraperitoneal administration	*	Sigma-Aldrich,USA	*	*	*	*	Received one gastric intubation of PQ(50mg/kg)	30min	CUR ST	Saline	*	14days	PaO2,HYP content
Hu, Y.	2018	China	Sprague–Dawley rats	220-240g	*	Male	3	3	CUR LPMPS	Inject intratracheally	LPMP	Guangfu Fine Chemical Institute of Tianjin	*	*	*	*	Received one intratracheal instillation of BLM(5mg/kg)	2days	CUR ST	*	*	7days or 28days	(BALF)TNF-α concentration(7thD),HYP content(28thD)
Chen, H.	2019	China	Wistar rats	224.24±4.36g	6 weeks old	*	18	17	CUR 200mg/kg	Intraperitoneal administration	*	Shanghai Baoman Bio-technology Co., Ltd., Shanghai, China	*	*	*	*	Received one gastric intubation of PQ(20mg/kg)	1hous	CUR ST	Saline	*	5days	PaO2
Gouda, M. M.	2020	India	C57BL/6 mice	25±5g	7–8 weeks old	Male	5	5	CUR 75mg/kg	Administer intraperitoneally	*	Himedia Laboratories Pvt. Bengaluru Ltd. (India)	*	*	*	*	Received one intranasal administeration of BLM (3 U/kg)	24hours	CUR ST	*	*	7days	BALF protein content
Barsan, M.	2021	Romania	Wistar rats	average weight of 320 g	*	Male	7	7	CUR 50mg/kg	Oral	*	Sigma-Aldrich,USA	≥80%	*	*	*	Received one intratracheal instillation of silica (3g/L)	10days	CUR 3times/week	*	110days	120days	(Lung tissue)MDA,(Blood)GSH
Durairaj, P.	2021	India	Wistar rats	325-350g	*	Male	6	6	CUR 300mg/kg	Gastric intubation	*	*	*	CUR QD	*	10days	Received one intravenous injection of BLM (7.5 U/kg)	*	CUR QD	*	7days	7days	(BALF)HYP content
Hemmati, A. A.	2021	Iran	Sprague–Dawley rats	170-220g	*	Male	10	10	NANOCUR 200 μg/kg	Nebulization inhalation	*	Merck (Concord Rd, Billerica MA)	*	*	*	*	Received one intratracheal instillation of BLM(5 IU/kg)	*	CUR QD	*	21days	21days	HYP content,(Lung tissue)TNF-α,TGF-β concentration
Hosseini, A.	2021	Iran	Wistar rats	180-250g	*	Male	6	6	NANOCUR 30mg/kg	Gastric intubation	*	Exir NanoSina Company, Tehran, Iran	*	*	*	*	Received one gastric intubation of PQ(5mg/kg)	*	CUR QD	*	7days	7days	MDA,HYP content
Miao, Y. M.	2021	China	ICR mice	22-25g	*	Female	6	6	CUR 75mg/kg	Rectal administration	*	Nanjing Jingzhu Biological Technology Co., Ltd. (Nanjing, China)	>98%	*	*	*	Received one intratracheal instillation of silica(100mg/kg)	*	CUR QD	*	30days	30days	HYP content
							6	6									Received one intratracheal instillation of BLM(5mg/kg)	*			21days	21days	HYP content
Kumari, S.	2022	India	Swiss Strain Mice	25-30g	*	Female	5	5	CUR 5mg/kg	Intranasal administration	Dimethyl Sulphoxide	Sigma-Aldrich,USA	*	CUR ST	DMSO	1hous	Received intranasal silica(200mg/mL) QOD	*	CUR QOD	DMSO	21days	22days	MPO activity,HYP content,(BALF)NO
