# Supplementary material for: Protective effects and mechanism of curcumin in animal models of pulmonary fibrosis: a preclinical systematic review and meta-analysis
Source: Front Pharmacol. 2023 Oct 13;14:1258885. doi: 10.3389/fphar.2023.1258885 (PMC10613035; doi:10.3389/fphar.2023.1258885)
Supplement: Supplementary file 2 [file DataSheet1.docx]

Protective effects and mechanism of Curcumin in animal models of pulmonary fibrosis: A preclinical systematic review and meta-analysis

Contents

Part 1 four electronic database searchable formats………………………………………………S2

Part 2 Sensitivity analysis…………………………………………………………….….……….S7

Part 3 Subgroup Analysis…………………………………………..………………………….….S8

Part 4 Publication bias ……………………………………………………………………………S9

**1 four electronic database searchable formats**

**1.1 Pubmed**

#1 ((((((((((Pulmonary Fibrosis[MeSH Terms]) OR (lung fibrosis[Title/Abstract])) OR (Fibrosis, Pulmonary[Title/Abstract])) OR (Pulmonary Fibroses[Title/Abstract])) OR (Fibroses, Pulmonary[Title/Abstract])) OR (Alveolitis, Fibrosing[Title/Abstract])) OR (Alveolitides, Fibrosing[Title/Abstract])) OR (Fibrosing Alveolitides[Title/Abstract])) OR (Fibrosing Alveolitis[Title/Abstract])) OR (Idiopathic Diffuse Interstital Pulmonary Fibrosis[Title/Abstract])

#2 ((((((((((((((((((Idiopathic Pulmonary Fibrosis[MeSH Terms] OR (Pulmonray Fibrosis, Idiopathic[Title/Abstract]) OR (Idiopathic Fibrosis Alveolitis, Chronic Form[Title/Abstract])) OR (Fibrosing Alveolitis, Cryptogenic[Title/Abstract])) OR (Fibrocystic Pulmonary Dysplasia[Title/Abstract])) OR (Dysplasia, Fibrocystic Pulmonary[Title/Abstract])) OR (Fibrocystic Pulmonary Dysplasias[Title/Abstract])) OR (Pulmonary Dysplasia, Fibrocystic[Title/Abstract])) OR (Cryptogenic Fibrosing Alveolitis[Title/Abstract])) ) OR (Cryptogenic Fibrosing Alveolitides[Title/Abstract])) OR (Fibrosing Alveolitides, Cryptogenic[Title/Abstract])) OR (Pulmonray Fibrosis, Idiopathic[Title/Abstract])) OR (Usual Interstitial Pneumonia[Title/Abstract] OR Interstitial Pneumonia, Usual[Title/Abstract])) OR (Usual Interstitial Pneumonias[Title/Abstract])) OR (Interstitial Pneumonias, Usual[Title/Abstract])) OR (Pneumonitides, Usual Interstitial[Title/Abstract])) OR (Pneumonitis, Usual Interstitial[Title/Abstract])) OR (Usual Interstitial Pneumonitides[Title/Abstract])) OR (Usual Interstitial Pneumonitis[Title/Abstract])) OR (Familial Idiopathic Pulmonary Fibrosis[Title/Abstract])) OR (Idiopathic Pulmonary Fibrosis, Familial[Title/Abstract])

#3 ((Acute Lung Injury[MeSH Terms]) OR (Respiratory Distress Syndrome[MeSH Terms])) OR ((((((((((((((((((Acute Lung Injuries[Title/Abstract]) OR (Lung Injuries, Acute[Title/Abstract])) OR (Lung Injury, Acute[Title/Abstract])) OR (Distress Syndrome, Respiratory[Title/Abstract])) OR (Distress Syndromes, Respiratory[Title/Abstract])) OR (Respiratory Distress Syndromes[Title/Abstract])) OR (Syndrome, Respiratory Distress[Title/Abstract])) OR (Shock Lung[Title/Abstract])) OR (Lung, Shock[Title/Abstract])) OR (Respiratory Distress Syndrome, Acute[Title/Abstract])) OR (Acute Respiratory Distress Syndrome[Title/Abstract])) OR (ARDS, Human[Title/Abstract])) OR (Human ARDS[Title/Abstract])) OR (Respiratory Distress Syndrome, Pediatric[Title/Abstract])) OR (Pediatric Respiratory Distress Syndrome[Title/Abstract])) OR (Respiratory Distress Syndrome, Adult[Title/Abstract])) OR (Adult Respiratory Distress Syndrome[Title/Abstract] )))

#4 #1 OR #2 OR #3

#5 (curcumin[MeSH Terms]) OR (((((((curcumin[Title/Abstract]) OR (curcuminoid[Title/Abstract])) OR (turmeric[Title/Abstract])) OR (curcuma[Title/Abstract])) OR (Meriva curcuma[Title/Abstract])) OR (Curcuma longa[Title/Abstract])) OR (Curcumas[Title/Abstract])) OR (turmeric[Title/Abstract])

#6 ("Animals" [Mesh Terms] OR "Models, Animal" [Mesh Terms] OR "Animals, Laboratory" [Mesh Terms] OR "Animal Experimentation" [Mesh Terms]) OR ("Animalia" [Title/Abstract] OR "Animal" [Title/Abstract] OR "Metazoa" [Title/Abstract] OR "Animal Model" [Title/Abstract] OR "Animal Models" [Title/Abstract] OR "Model, Animal" [Title/Abstract] OR "Laboratory Animal Models" [Title/Abstract] OR "Animal Model, Laboratory" [Title/Abstract] OR "Animal Models, Laboratory" [Title/Abstract] OR "Laboratory Animal Model" [Title/Abstract] OR "Model, Laboratory Animal" [Title/Abstract] OR "Experimental Animal Models" [Title/Abstract] OR "Animal Model, Experimental" [Title/Abstract] OR "Animal Models, Experimental" [Title/Abstract] OR "Experimental Animal Model" [Title/Abstract] OR "Model, Experimental Animal" [Title/Abstract] OR "Models, Experimental Animal" [Title/Abstract] OR "Experimentation, Animal" [Title/Abstract] OR "Animal Research" [Title/Abstract] OR "Research, Animal" [Title/Abstract] OR "Animal Experimental Use" [Title/Abstract] OR "Animal Experiments" [Title/Abstract] OR "Animal Experiment" [Title/Abstract] OR "Experiment, Animal" [Title/Abstract] OR "Experiments, Animal" [Title/Abstract] OR "Animal, Laboratory" [Title/Abstract] OR "Laboratory Animal" [Title/Abstract] OR "Laboratory Animals" [Title/Abstract] OR "preclinical studies" [Title/Abstract] OR "experimental animals" [Title/Abstract] OR "experimental animal" [Title/Abstract])

#7 #4 AND #5 AND #6

**1.2 Embase**

#1 'pulmonary fibrosis'/exp OR 'idiopathic pulmonary fibrosis'/exp

#2 'lung fibrosis':ab,ti OR 'fibrosis, pulmonary':ab,ti OR 'pulmonary fibroses':ab,ti OR 'fibroses, pulmonary':ab,ti OR 'alveolitis, fibrosing':ab,ti OR 'alveolitides, fibrosing':ab,ti OR 'fibrosing alveolitides':ab,ti OR 'fibrosing alveolitis':ab,ti OR 'idiopathic diffuse interstital pulmonary fibrosis':ab,ti OR 'idiopathic fibrosis alveolitis, chronic form':ab,ti OR 'fibrosing alveolitis, cryptogenic':ab,ti OR 'fibrocystic pulmonary dysplasia':ab,ti OR 'dysplasia, fibrocystic pulmonary':ab,ti OR 'fibrocystic pulmonary dysplasias':ab,ti OR 'pulmonary dysplasia, fibrocystic':ab,ti OR 'cryptogenic fibrosing alveolitis':ab,ti OR 'cryptogenic fibrosing alveolitides':ab,ti OR 'fibrosing alveolitides, cryptogenic':ab,ti OR 'pulmonray fibrosis, idiopathic':ab,ti OR 'usual interstitial pneumonia':ab,ti OR 'interstitial pneumonia, usual':ab,ti OR 'usual interstitial pneumonias':ab,ti OR 'interstitial pneumonias, usual':ab,ti OR 'pneumonitides, usual interstitial':ab,ti OR 'pneumonitis, usual interstitial':ab,ti OR 'usual interstitial pneumonitides':ab,ti OR 'usual interstitial pneumonitis':ab,ti OR 'familial idiopathic pulmonary fibrosis':ab,ti OR 'idiopathic pulmonary fibrosis, familial':ab,ti

#3 'acute lung injury'/exp OR 'adult respiratory distress syndrome'/exp

#4 'acute lung injuries':ab,ti OR 'lung injuries, acute':ab,ti OR 'lung injury, acute':ab,ti OR 'distress syndrome, respiratory':ab,ti OR 'distress syndromes, respiratory':ab,ti OR 'respiratory distress syndromes':ab,ti OR 'syndrome, respiratory distress':ab,ti OR 'shock lung':ab,ti OR 'lung, shock':ab,ti OR 'respiratory distress syndrome, acute':ab,ti OR 'acute respiratory distress syndrome':ab,ti OR 'ards, human':ab,ti OR 'human ards':ab,ti OR 'respiratory distress syndrome, pediatric':ab,ti OR 'pediatric respiratory distress syndrome':ab,ti OR 'respiratory distress syndrome, adult':ab,ti OR 'adult respiratory distress syndrome':ab,ti

#5 #1 OR #2 OR #3 OR #4

#6 'curcumin'/exp

#7 curcumin:ab,ti OR curcuminoid:ab,ti OR curcuma:ab,ti OR 'meriva curcuma':ab,ti OR 'curcuma longa':ab,ti OR curcumas:ab,ti OR turmeric:ab,ti

#8 #6 OR #7

#9 'animals'/exp OR 'models, animal'/exp OR 'animals, laboratory'/exp OR 'animal experimentation'/exp

#10 animalia:ab,ti OR animal:ab,ti OR metazoa:ab,ti OR 'animal model':ab,ti OR 'animal models':ab,ti OR 'model, animal':ab,ti OR 'laboratory animal models':ab,ti OR 'animal model, laboratory':ab,ti OR 'animal models, laboratory':ab,ti OR 'laboratory animal model':ab,ti OR 'model, laboratory animal':ab,ti OR 'experimental animal models':ab,ti OR 'animal model, experimental':ab,ti OR 'animal models, experimental':ab,ti OR 'experimental animal model':ab,ti OR 'model, experimental animal':ab,ti OR 'models, experimental animal':ab,ti OR 'experimentation, animal':ab,ti OR 'animal research':ab,ti OR 'research, animal':ab,ti OR 'animal experimental use':ab,ti OR 'animal experiments':ab,ti OR 'animal experiment':ab,ti OR 'experiment, animal':ab,ti OR 'experiments, animal':ab,ti OR 'animal, laboratory':ab,ti OR 'laboratory animal':ab,ti OR 'laboratory animals':ab,ti OR 'preclinical studies':ab,ti OR 'experimental animals':ab,ti OR 'experimental animal':ab,ti

#11 #9 OR #10

#12 #5 AND #8 AND #11

**1.3 Web of science**

#1 TS=(Idiopathic Pulmonary Fibrosis OR Pulmonary Fibrosis)

#2 AB=(Pulmonray Fibrosis, Idiopathic OR Idiopathic Fibrosis Alveolitis, Chronic Form OR Fibrosing Alveolitis, Cryptogenic OR Fibrocystic Pulmonary Dysplasia OR Dysplasia, Fibrocystic Pulmonary OR Fibrocystic Pulmonary Dysplasias OR Pulmonary Dysplasia, Fibrocystic OR Cryptogenic Fibrosing Alveolitis OR Cryptogenic Fibrosing Alveolitides OR Fibrosing Alveolitides, Cryptogenic OR Pulmonray Fibrosis, Idiopathic OR Usual Interstitial Pneumonia OR Interstitial Pneumonia, Usual OR Usual Interstitial Pneumonias OR Interstitial Pneumonias, Usual OR Pneumonitides, Usual Interstitial OR Pneumonitis, Usual Interstitial OR Usual Interstitial Pneumonitides OR Usual Interstitial Pneumonitis OR Familial Idiopathic Pulmonary Fibrosis OR Idiopathic Pulmonary Fibrosis, Familial OR lung fibrosis OR Fibrosis, Pulmonary OR Pulmonary Fibroses OR Fibroses, Pulmonary OR Alveolitis, Fibrosing OR Alveolitides, Fibrosing OR Fibrosing Alveolitides OR Fibrosing Alveolitis OR Idiopathic Diffuse Interstital Pulmonary Fibrosis)

#3 TS= (Acute Lung Injury OR Respiratory Distress Syndrome)

#4 AB=(Acute Lung Injuries OR Lung Injuries, Acute OR Lung Injury, Acute OR Distress Syndrome, Respiratory OR Distress Syndromes, Respiratory OR Respiratory Distress Syndromes OR Syndrome, Respiratory Distress OR Shock Lung OR Lung, Shock OR Respiratory Distress Syndrome, Acute OR Acute Respiratory Distress Syndrome POR ARDS, Human OR Human ARDS OR Respiratory Distress Syndrome, Pediatric OR Pediatric Respiratory Distress Syndrome OR Respiratory Distress Syndrome, Adult OR Adult Respiratory Distress Syndrome )

#5 #1 OR #2 OR #3 OR #4

#6 TS=(curcumin)

#7 AB=(curcumin OR curcuminoid OR turmeric OR curcuma OR Meriva curcuma OR Curcuma longa OR Curcumas OR turmeric)

#8 #6 OR #7

#9 TS=(Animals OR Models, Animal OR Animals, Laboratory OR Animal Experimentation )

#10 AB= (Animalia OR Animal OR Metazoa OR Animal Model OR Animal Models OR Model, Animal OR Laboratory Animal Models OR Animal Model, Laboratory OR Animal Models, Laboratory OR Laboratory Animal Model OR Model, Laboratory Animal OR Experimental Animal Models OR Animal Model, Experimental OR Animal Models, Experimental OR Experimental Animal Model OR Model, Experimental Animal OR Models, Experimental Animal OR Experimentation, Animal OR Animal Research OR Research, Animal OR Animal Experimental Use OR Animal Experiments OR Animal Experiment OR Experiment, Animal OR Experiments, Animal OR Animal, Laboratory OR Laboratory Animal OR Laboratory Animals OR preclinical studies OR experimental animals OR experimental animal )

#11 #9 OR #10

#12 #5 AND #8 AND #11

**1.4 Cochrane Library**

#1 MeSH descriptor: [Animals] explode all trees

#2 MeSH descriptor: [Models, Animal] explode all trees

#3 MeSH descriptor: [Animals, Laboratory] explode all trees

#4 (Animalia OR Animal OR Metazoa OR Animal Model OR Animal Models OR Model, Animal OR Laboratory Animal Models OR Animal Model, Laboratory OR Animal Models, Laboratory OR Laboratory Animal Model OR Model, Laboratory Animal OR Experimental Animal Models OR Animal Model, Experimental OR Animal Models, Experimental OR Experimental Animal Model OR Model, Experimental Animal OR Models, Experimental Animal OR Experimentation, Animal OR Animal Research OR Research, Animal OR Animal Experimental Use OR Animal Experiments OR Animal Experiment OR Experiment, Animal OR Experiments, Animal OR Animal, Laboratory OR Laboratory Animal OR Laboratory Animals OR preclinical studies OR experimental animals OR experimental animal):ti,ab,kw (Word variations have been searched)

#5 MeSH descriptor: [Animal Experimentation] explode all trees

#6 #1 OR #2 OR #3 OR #4 OR #5

#7 MeSH descriptor: [curcumin] explode all trees

#8 (curcumin OR curcuminoid OR turmeric OR curcuma OR Meriva curcuma OR Curcuma longa OR Curcumas OR turmeric):ti,ab,kw (Word variations have been searched)

#9 #7 OR #8

#10 MeSH descriptor: [Pulmonary Fibrosis] explode all trees

#11 MeSH descriptor: [Idiopathic Pulmonary Fibrosis] 1 tree(s) exploded

#12 MeSH descriptor: [Acute Lung Injury] explode all trees

#13 MeSH descriptor: [Respiratory Distress Syndrome] explode all trees

#14 (Pulmonray Fibrosis, Idiopathic OR Idiopathic Fibrosis Alveolitis, Chronic Form OR Fibrosing Alveolitis, Cryptogenic OR Fibrocystic Pulmonary Dysplasia OR Dysplasia, Fibrocystic Pulmonary OR Fibrocystic Pulmonary Dysplasias OR Pulmonary Dysplasia, Fibrocystic OR Cryptogenic Fibrosing Alveolitis OR Cryptogenic Fibrosing Alveolitides OR Fibrosing Alveolitides, Cryptogenic OR Pulmonray Fibrosis, Idiopathic OR Usual Interstitial Pneumonia OR Interstitial Pneumonia, Usual OR Usual Interstitial Pneumonias OR Interstitial Pneumonias, Usual OR Pneumonitides, Usual Interstitial OR Pneumonitis, Usual Interstitial OR Usual Interstitial Pneumonitides OR Usual Interstitial Pneumonitis OR Familial Idiopathic Pulmonary Fibrosis OR Idiopathic Pulmonary Fibrosis, Familial):ti,ab,kw (Word variations have been searched)

#15 (lung fibrosis OR Fibrosis, Pulmonary OR Pulmonary Fibroses OR Fibroses, Pulmonary OR Alveolitis, Fibrosing OR Alveolitides, Fibrosing OR Fibrosing Alveolitides OR Fibrosing Alveolitis OR Idiopathic Diffuse Interstital Pulmonary Fibrosis):ti,ab,kw (Word variations have been searched)

#16 (Acute Lung Injuries OR Lung Injuries, Acute OR Lung Injury, Acute OR Distress Syndrome, Respiratory OR Distress Syndromes, Respiratory OR Respiratory Distress Syndromes OR Syndrome, Respiratory Distress OR Shock Lung OR Lung, Shock OR Respiratory Distress Syndrome, Acute OR Acute Respiratory Distress Syndrome POR ARDS, Human OR Human ARDS OR Respiratory Distress Syndrome, Pediatric OR Pediatric Respiratory Distress Syndrome OR Respiratory Distress Syndrome, Adult OR Adult Respiratory Distress Syndrome ):ti,ab,kw

#17 #10 OR #11 OR #12 OR #13 OR #14 OR #15 OR #16

#18 #6 AND #9 AND #17

**2** **Sensitivity analysis**


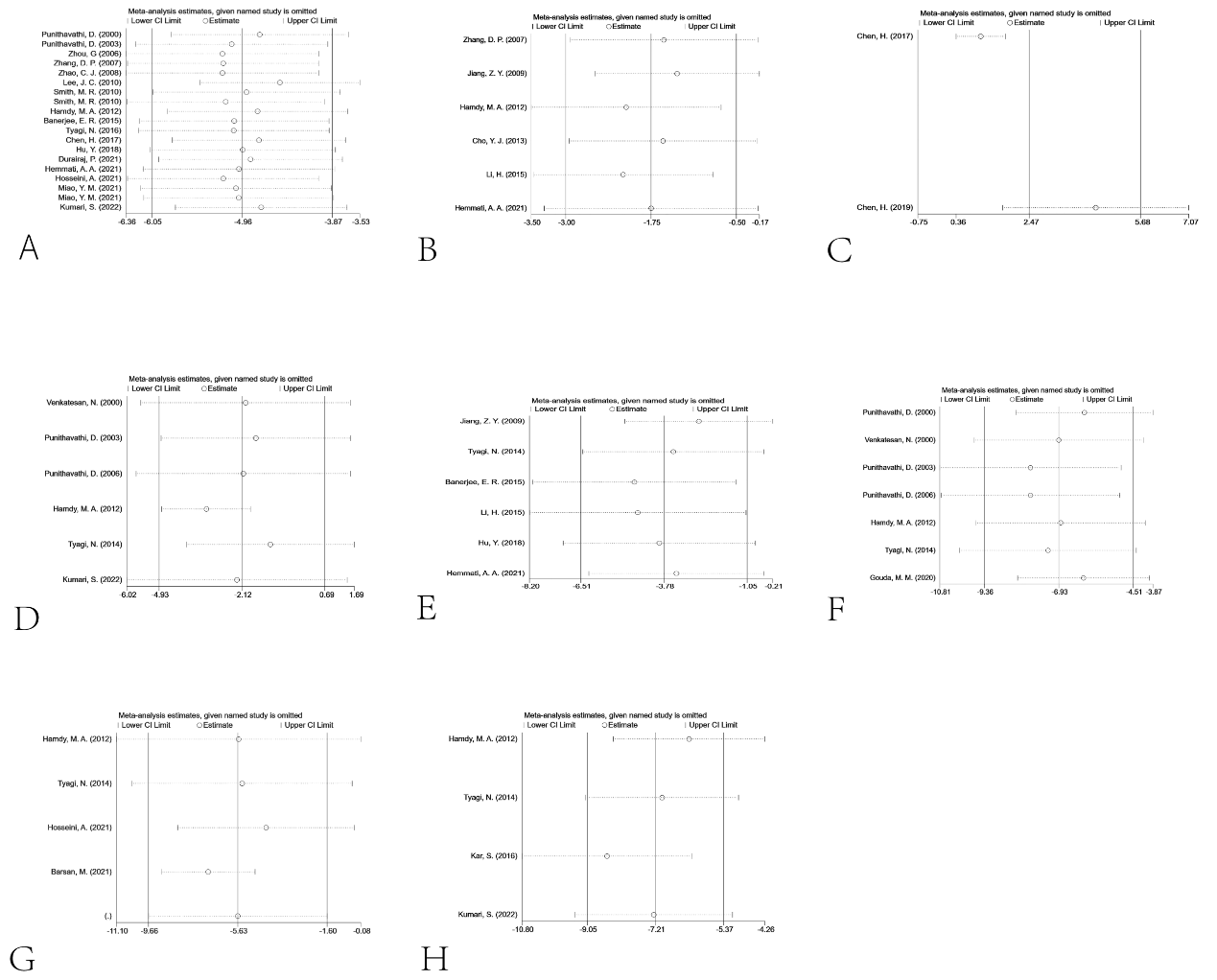


Figure1 Sensitivity analysis

(A–H) represent the sensitivity of HYP content, TGF-β concentration, PaO2, MPO activity, TNF-α concentration, BALF protein content , MDA and NO, respectively. The sensitivity analysis was conducted by omitting single studies one by one, and no study with critical influence was found.

**3 Subgroup Analysis**

**
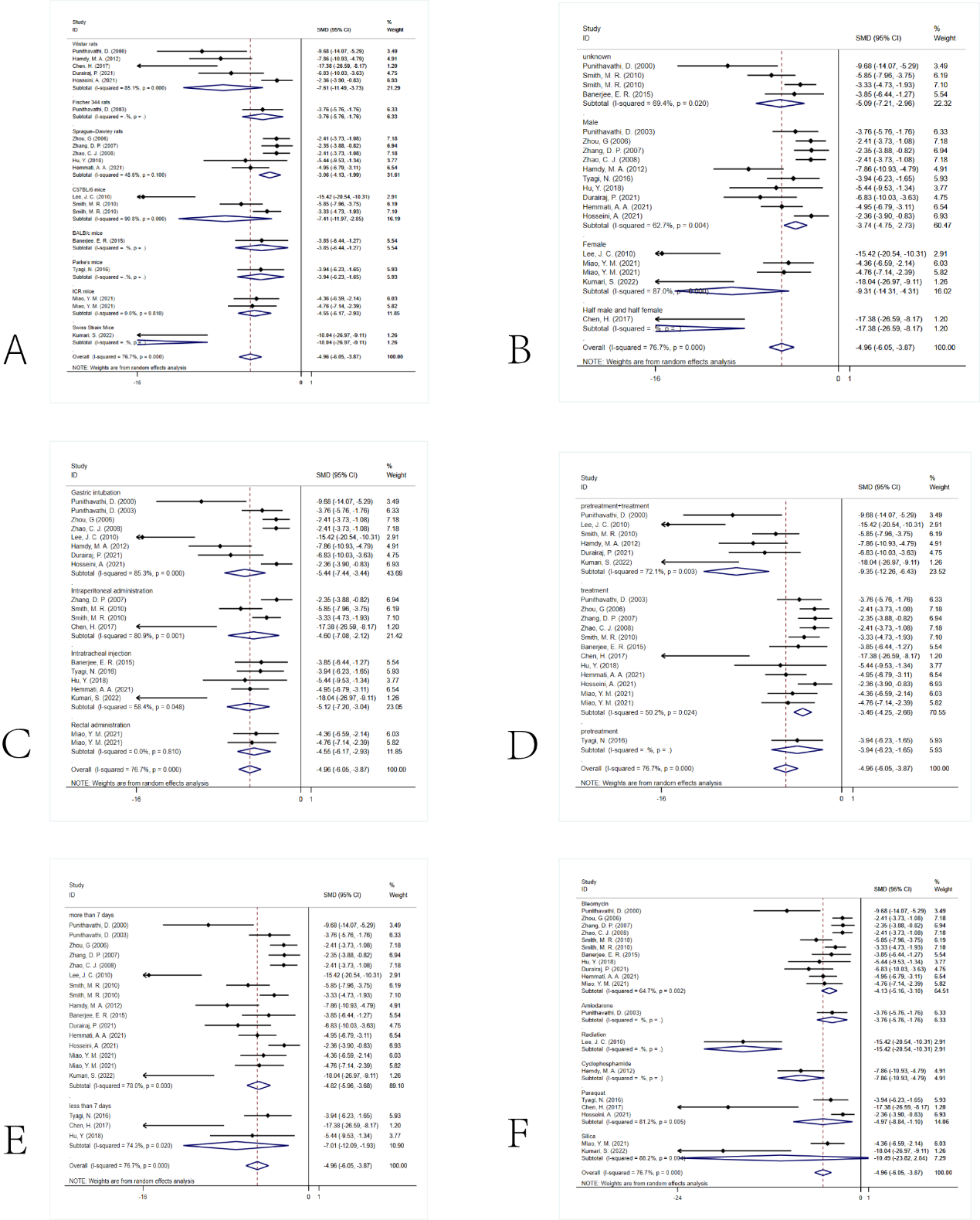
**

Figure2 Subgroup Analysis

(A–F) represent the subgroup of animal species, animal sex, route of administration, intervention time point, duration of treatment and modelling approach. The results suggest that animal species and route of administration may be a source of heterogeneity in HYP content.

**4 Publication bias**


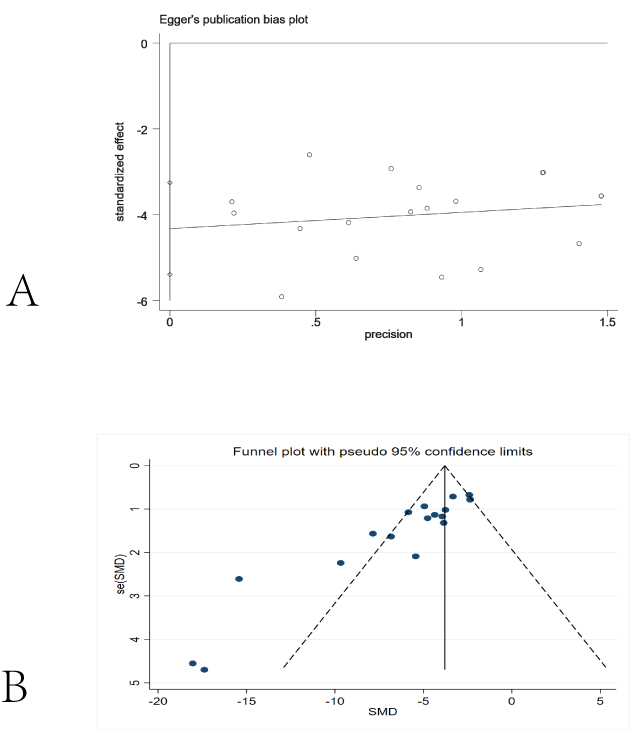


Figure3 Publication bias.

(A) Egger’s test of HYP content. *p*=0.000, t =-7.97. (B) Funnel plot of HYP content. (A) and (B) indicated that there was publication bias.
